# Assessing educational poverty: Insights into youth opportunities

**DOI:** 10.1371/journal.pone.0346156

**Published:** 2026-05-18

**Authors:** Cristina Davino, Antonio De Falco, Rosa Fabbricatore, Rosaria Romano

**Affiliations:** 1 Department of Economics and Statistics, University of Naples Federico II, Naples, Italy; 2 Department of Sociology and Social Research, University of Milano-Bicocca, Milan, Italy; University of Salerno, ITALY

## Abstract

Educational opportunities (EO) are the main drivers for ensuring the balanced growth of future generations and a more prosperous future for the community. A lack or inadequacy of EO can lead to conditions of educational poverty. Despite its fundamental relevance, measuring this phenomenon is still an open challenge in the scientific community, given its complex and multidimensional nature and multiple connections with other aspects such as family background and cognitive and emotional development. The objective of this paper is to identify the main factors that may contribute to measuring educational poverty in terms of deprivation or lack of opportunities for youth aged 15–19. A pilot study of youth living in the city of Naples is proposed to test a conceptual model and to characterize groups of youth with different levels of educational poverty. Factor analysis was carried out to uncover the underlying dimensions regarding EO. The findings underscore the phenomenon’s three-dimensional aspect, encompassing the dimensions of *Family*, *School*, and *Environment*. Additionally, the study revealed the diversity among respondents about these three dimensions by exploiting a hierarchical cluster analysis. Results showed that family background, Italian grade, gender, and type of school are significantly related to the detected groups, providing valuable insights into the issue of educational poverty.

## 1 Introduction

The notion of educational poverty (EP) emerged in the late 1990s within the social sciences, when a new understanding of poverty based on the recognition of its multidimensional nature led to a new phase of research [1]. Sen’s capability approach provided a theoretical foundation for multidimensional poverty analysis [2]. According to Sen, poverty and individuals’ well-being cannot be solely understood in terms of income or material deprivation; it is also essential to consider the capabilities and opportunities that individuals have access to, as these enable them to pursue what they consider valuable for their lives. Within this framework, education emerges as a distinct dimension of poverty since it is regarded as a crucial resource that enhances individuals’ capability to achieve [3]. Deprivation of education is particularly insidious as it not only leads to a lack of human capital but also significantly hinders individual opportunities for personal development and fulfilment. This, in turn, impacts social inclusion and gives rise to long-term consequences that jeopardize individuals’ futures, contributing to the persistence of inequalities fueling the intergenerational transmission of disadvantage [4–6]. Since EP compromises the present and future of individuals, particularly adolescents, leading to negative consequences for the whole society, the topic has gained institutional and political attention in recent times.

In 2021, the European Union adopted the *Child Guarantee program*, aimed at reducing child poverty by ensuring access to quality education, early childhood services, healthcare, and adequate nutrition for all European children and adolescents [7]. This measure aligns with the United Nations’ 2030 Agenda for Sustainable Development, emphasising inclusive and equitable education and lifelong learning opportunities for all. In the Italian context, significant strides have been made in addressing EP after a series of legislative interventions primarily focused on safeguarding the welfare of children and adolescents (see, for example, the establishment of the National Observatory for Children and Adolescents via Law 451/1997). In 2016, a fund was created to address child EP, supporting initiatives to overcome economic, social, and cultural barriers to quality education. More recently, in 2022, the National Plan for Recovery and Resilience (PNRR) funded socio-educational projects by Third Sector entities, highlighting EP as a central issue on the national political agenda [8].

Like other complex phenomena, EP encompasses various unreadily observable dimensions, posing challenges in comprehensively defining and measuring it. How a phenomenon is conceptualized and measured can influence its recognition, extent, and severity, shaping policy decisions and strategies to mitigate vulnerability, particularly when addressing issues with significant social impact. Consequently, efforts to define and measure EP should closely align with policy interventions.

Recently, a new perspective on the phenomenon views EP as the lack or inadequacy of educational opportunities (EO) available to individuals within their living environment, which significantly hinders their ability to develop cognitive, cultural, and social skills [4].

In broader terms, EO refer to access to and availability of resources and services that enable individuals to develop skills, knowledge, and abilities necessary for their personal and professional development. They include not only learning opportunities that occur within the formal educational system [9] but also extend to a wide range of educational, cultural and social experiences outside of formal education [10,11]. These experiences are crucial for cognitive development and the acquisition of social skills and relationships, particularly for children and young people [12,13]. Inequalities in access to EO are a persistent problem affecting individuals’ lives and society’s development, raising issues of equity and social justice [14]. A recent proposal by a scientific commission from ISTAT, presented at the *fifteenth National Conference of Statistics* in Rome on 3–4 July 2024, introduces new indicators to measure educational poverty (EP) using a multidimensional approach: EP, targeting ages 0–19, is divided into educational resources and children’s outcomes.

While the lack or insufficiency of EO also affects the adult population, the primary focus regarding resource inequalities is on youth and children. This is because they are at a critical development stage, where EO has long-lasting impacts on their future cognitive and social-emotional development [15]. Addressing educational inequalities during childhood and adolescence is crucial for breaking cycles of disadvantage and promoting a more equitable society. Research highlights that disparities in learning opportunities mainly affect individuals from disadvantaged socio-economic backgrounds [11,16]. Moreover, the long-term effects of unequal EO at an early age extend beyond academic achievement, encompassing reduced employability, lower salaries, and a higher likelihood of experiencing social exclusion later in life [17].

The literature has identified several factors contributing to EO inequalities among young people, including socioeconomic background, parental education level, quality of schools, and living environment conditions. Students from higher socioeconomic backgrounds often benefit from better access to educational resources available at home and participation in extracurricular activities [18,19]. Similarly, parental education level and involvement in their children’s education are critical factors. Students whose parents have a higher level of education and are actively involved in their education tend to have better performance and school achievement [20,21]. The quality of the school environment, such as the availability of adequate facilities and a lower pupil-teacher ratio, significantly affects EO. Schools in disadvantaged areas often lack these resources, resulting in lower educational outcomes for students [22]. Additionally, the role played by school quality in students’ achievement seems more vital for students from poorer family backgrounds [16]. Broader environmental factors, such as the availability of educational and recreational facilities, such as libraries, theatres, museums, green areas, and community centres, as well as exposure to poverty and deprivation can influence children’s development and impact learning opportunities [23,24].

Starting from this premise, the work proposes a comprehensive model for measuring EP in terms of EO. This study is part of the Measuring and Mapping Poverty Education project, supported by the University Research Funding Program (FRA) of the University of Naples Federico II for 2023–2025.

We developed a conceptual framework defining EP as a latent, multidimensional construct measured across family, school, and environment dimensions. Based on existing literature, we proposed a model with two main hypotheses [25]: (1) students’ social backgrounds, including parental education and occupation, significantly influence educational deprivation; (2) EP, marked by limited access to educational opportunities (EO) in family, school, and environmental contexts, impacts cognitive (e.g., school performance) and non-cognitive abilities (e.g., self-esteem, motivation). In the present contribution, we focus primarily on the first hypothesis and on the operationalization of EP in terms of lack of EO. The second hypothesis, concerning the relationship between EO and cognitive and non-cognitive outcomes, is only partially explored and will be investigated more systematically in future studies. The paper reports the results of a pilot study on high school students aged 15–19 in Naples and its suburbs, focusing on EO dimensions and their links to personal and family backgrounds.

By doing so, our proposal aims to capture the multidimensional and latent nature of EP. Methodological challenges in measuring EP, such as data availability, target population selection, and indicator choice, pose significant obstacles to developing a standardized framework. Despite advancements in understanding EP, there remains a need for more comprehensive and nuanced research to establish a unified theoretical and methodological approach. Addressing gaps in knowledge, including understanding the interplay between EP and individual or contextual factors, and exploring innovative measurement strategies are crucial steps toward making significant advances in this field of research.

The paper is organized as follows. Section 2 reviews the concept of EP, tracing its evolution from initial academic discussions to more structured, recent frameworks. It also presents the dimensions and indicators of EP, distinguishing between unidimensional approaches, which focus on single indicators, and multidimensional frameworks encompassing a more comprehensive range of socio-economic, emotional, and environmental factors. Section 3 illustrates the conceptual framework adopted in this work, in which EP is defined as a latent, multidimensional construct and operationalised through specific dimensions and indicators. Section 4 and Section 5 describe the data and methodology, including the data collection process and statistical methods used to test the proposed model on a sample of high school students from Naples (Italy). Section 6 outlines the results of the statistical analysis, while Section 7 summarizes the essential findings and offers concluding remarks.

## 2 Understanding educational poverty

### 2.1 A review of definitions and key concepts

The concept of EP was first introduced by Checchi [26], who coined the expression “povertà di istruzione” (poverty of education) to highlight the critical role of education in perpetuating poverty and inequalities in modern societies. In this regard, the absence of educational credentials can lead to a dual condition of deprivation, encompassing the lack of human capital in its instrumental function (such as access to employment and income) and the disempowering of functional capabilities, significantly impacting individuals’ relational capacity. Allmendinger [27] expanded the concept by defining EP as an unacceptably low level of education within a specific society, emphasizing its impact on individuals’ living conditions. Later, Allmendinger and Leibfried [28] further developed EP by incorporating the lack of educational certificates and competencies into its definition. Lohmann and Ferger [3] framed EP as a measure of educational attainment and as a normative concept that refers to the thresholds of acceptable poverty levels in a given society.

Additional studies from Italian and European contexts have often associated EP with deficiencies in education, such as low educational qualifications or skills. For example, Barbieri and Cipollone [29] define EP as a condition marked by inadequate levels of basic skills, including issues related to reading, comprehension, writing and difficulties in performing elementary mathematical operations. In a recent study, Giancola and Salmieri [30] adopted a definition of EP based on two key indicators: 1) education levels below upper secondary school and 2) low levels of basic skills. This definition encompasses various forms of EP, including individuals without a secondary school diploma, those with diplomas but lacking basic skills, and those with both low educational attainment and skill deficiencies. The authors focus on fundamental skills such as reading, comprehension, writing, numeracy, and problem-solving, which are assessed through international surveys like PISA and PIAAC. They argue that lacking these skills not only leads to EP but also hinders economic and social inclusion.

While existing definitions of EP emphasize the absence of educational credentials or inadequate acquisition of basic skills across all age groups, it is increasingly recognized that EP significantly impacts individuals during their formative years, underscoring the pivotal role of educational processes in childhood and adolescence [31]. In this regard, a significant step towards a more comprehensive definition of EP was taken by the NGO Save the Children with the publication of the report *La lampada di Aladino* [32]. EP is described as ‘the deprivation of children and adolescents from the opportunity to learn, experience, develop, and freely cultivate their capabilities, talents, and aspirations’ (p. 4).

Inspired by Sen’s capabilities theory and Nussbaum’s development framework [33], Save the Children’s definition encompasses four specific dimensions of educational deprivation [34]: 1) *learning to understand*, which is related to cognitive and problem-solving skills; 2) *learning to be*, which involves psychological and emotional development; 3) *learning to live together*, that is, the ability to promote social and inter-personal relationships; 4) *learning to lead an autonomous and active life* involves aspects like health, physical integrity, and food security, regarded as functional conditions for education and other learning opportunities. Within this framework, EP is seen as a deprivation of these learning chances and a loss of human capital in its instrumental function [13].

Remarkably, Save the Children introduced a novel conceptualization of the phenomenon by proposing a definition of EP centred on the lack of access to EO that hinders personal development. This deprivation affects cultural resources and cognitive abilities and impacts emotional, relational, and life-planning aspects. These factors, along with cognitive abilities, shape the developmental trajectory of minors [35]. Save the Children’s definition aligns with a multidimensional approach and highlights the interconnected relationship between economic poverty and EP. Growing up in a disadvantaged family environment and lacking developmental opportunities from an early age often imposes a significant discriminatory burden on minors compared to their peers, with consequences that may become irreversible over time. In a vicious cycle, EP exacerbates economic poverty and vice versa [32].

### 2.2 Dimensions and indicators of EP

Building upon the definitions of EP outlined in the previous section, a primary distinction can be made between studies that operationalize the phenomenon by considering a single dimension of analysis and those that, acknowledging its multifaceted nature, incorporate multiple dimensions of analysis.

#### 2.2.1 Unidimensional approaches.

Checchi [26] associates EP primarily with education and develops a measurement approach centred on educational qualifications. Non-attainment of the minimum educational level, such as compulsory schooling, is a marker of EP. Checchi distinguishes between absolute and relative EP, advocating for an approach grounded in absolute deprivation, where failure to reach a certain education threshold indicates EP. This lack of basic knowledge diminishes individual functional capacities, exacerbating inequality in levels of well-being experienced by a population. Similarly, Allmendinger [27] adopts a unidimensional approach grounded in an absolute conception of EP, defining a poverty line based on the attainment of qualifications corresponding to compulsory schooling. Subsequently, Allmendinger and Leibfried [28] further develop this approach by integrating competencies alongside qualifications, utilizing OECD PISA data to target 15-year-olds. They distinguish between credential-based EP, which indicates the absence of educational certificates, and competency-based EP, which relates to minimal skills essential for economic and social participation. They also differentiate between absolute and relative measures of EP. Lohman and Ferger [3], aligning with Checchi and Allmendinger, suggest considering the educational level attained and defining the minimum acceptable qualification within a society as a threshold for EP. They propose using a relative measure of EP in developed countries and an absolute measure where a significant portion of the population lacks formal education. This approach, employing indicators of education achieved (such as years of schooling or degree attained) or competencies, is also utilized in various other studies [5,36–39]. Moreover, studies have also explored socio-economic factors contributing to educational disadvantage, including socio-economic status and immigrant background [6,30,38,40] advocate for a measurement strategy based on indicators related to both educational achievements and deficiencies in essential competencies (such as literacy, numeracy, and basic mathematical operations, as well as personal and civic competence), crucial for individuals’ full development. This operationalization is considered crucial, as mere completion of compulsory education does not guarantee the possession, maintenance, and effective utilization of basic capabilities over time.

#### 2.2.2 Multidimensional approaches.

In the Italian context, Save the Children [32,41], in collaboration with the Scientific Committee on Educational Poverty in Italy and ISTAT, developed a multidimensional EP Index (EPI), designed to investigate the phenomenon’s intensity and allow territorial comparisons at the regional level. The EPI comprises 14 indicators, categorized into two sub-indices: the first includes measures associated with the school context and the provision of educational services, while the second covers indicators related to children’s participation in extracurricular activities. Later, Save the Children released revised editions of the EPI in 2016 [42] and 2018 [43] to monitor and compare regional EP trends. According to the rationale underlying the construction of the index, EP involves the deprivation of cognitive and non-cognitive skills such as motivation, self-esteem, cooperation, and empathy, which are equally fundamental. Operationally, EP is seen as a latent phenomenon where resources offered by social, cultural, and environmental contexts affect children’s EO. EP manifests primarily through two outcomes: the development of cognitive skills and non-cognitive skills, often referred to as social-emotional skills, which include aspects such as self-awareness, emotion management, stress management, empathy, effective communication, and effective relationships.

Building on this pioneering work, a team of researchers from the University of Pisa, the Italian Institute of Statistics (ISTAT), and the social enterprise ’Con i Bambini’ further advanced the development of a multidimensional EP measure [13]. Drawing upon the theoretical framework defined by Save the Children [34], ISTAT [44] formulated an EPI for individuals aged 15–29, which provides EP estimates at the Italian regional level. The index was developed using a set of indicators corresponding to four dimensions: 1) *participation*, denoting a deficiency/lack of participating in social life; 2) *resilience*, signifying a deficiency/lack in developing the ability to trust oneself and one’s abilities; 3) *standard of living*, indicating a deficiency/lack of opportunity to lead an inclusive, healthy and safe life; 4) *friendships and skills*, referring to a deficiency/lack in relating to others and skills needed to succeed in a fast-changing world.

In conclusion, the diverse definitions and approaches to measuring EP reflect its complex and multifaceted nature. From unidimensional approaches that focus on educational qualifications to multidimensional frameworks that consider socioeconomic and cultural factors, there is still no consensus on how to define and measure EP effectively. This plurality of research approaches is rooted in distinct theoretical traditions. From a human-capital perspective, education is primarily valued as an effective means to address social inequalities due to its instrumental returns in terms of employment and income, which encourages outcome-oriented operationalizations centered on qualifications and competencies. By contrast, the capability approach shifts attention toward the “conditions of possibility” that enable learning and development. Accordingly, a first strand of research defines EP mainly through educational attainment or minimum competencies (e.g., attainment below a given threshold, low literacy or numeracy), offering clear benchmarks and strong comparability, yet capturing deprivation ex post and risking a blurring of the distinction between contextual constraints that limit opportunities and the performance deficits that result from them. A second strand conceptualizes EP as a deprivation of educational resources, focusing upstream on the family, school, and environmental contexts that shape developmental trajectories. This distinction is not merely classificatory but has direct implications for operationalization: unidimensional strategies enhance interpretability through explicit poverty lines but may overlook the cumulative and interrelated nature of educational deprivation, whereas multidimensional, opportunity-based frameworks better reflect the complexity of EP while requiring critical decisions regarding the selection of relevant domains, the choice of appropriate indicators, and their conceptual roles (e.g., distinguishing inputs from outcomes).

## 3 Conceptual background

The literature review on EP highlights the evolution of its conceptualization, moving from unidimensional measures focused on educational attainment to more nuanced, multidimensional approaches. These frameworks increasingly emphasize the importance of examining various aspects of deprivation that extend beyond formal schooling to include other facets of human development. Within this context, the concept of EO has become central to understanding EP, conceptualized as a condition where individuals, particularly young people, are denied the chance to develop essential capabilities due to inadequate educational, social, and environmental opportunities.

Building on this theoretical framework, our approach seeks to operationalize EO as a critical measure of EP by identifying specific domains where deprivation occurs. While prior research has often examined EO holistically or through broad measures, our contribution lies in systematically identifying and categorizing the distinct spheres where these opportunities manifest. Specifically, we propose an analytical approach that draws from the definition put forth by Save the Children [32], which defines EP as the lack of opportunities for children and adolescents to learn, experience, grow, and nourish their skills, talents, and dreams. Our model distinguishes EO across three main dimensions: *Family*, *School*, and *Environment*. This conceptual framework allows for a more granular examination of how EO – or their absence – are distributed across the various contexts that shape children’s and adolescents’ development.

Each dimension is assessed through indicators that track the availability of educational opportunities for young people. For the identification of these indicators, we relied on two primary sources: the Save the Children report (2014) and the 2023 ISTAT survey “Bambini e ragazzi: comportamenti, atteggiamenti e progetti futuri”. Additional indicators were developed ad hoc to capture specific aspects of EP not addressed by these sources. Each indicator measures whether specific educational resources, experiences, or support systems are accessible to young individuals.

The *Family* dimension captures the resources within the household that support learning, such as access to educational materials and opportunities for cultural and extracurricular activities. The *School* dimension examines the resources provided by educational institutions, including digital tools and extracurricular programs. Lastly, the *Environment* dimension assesses the presence of community resources, such as public libraries, parks, and cultural institutions, that contribute to a child’s development outside of formal education.

Precisely, for the *Family* dimension, the indicators evaluate access to books and educational materials, the availability of time and space for study (including access to a desk, internet, and devices), parental support for schoolwork, opportunities for cultural enrichment (such as visits to museums, theatres, and concerts), and participation in hobbies and sports. The *School* dimension comprises indicators that assess the availability of digital tools for learning, use of libraries for study, access to remedial or advanced courses, participation in after-school programs (e.g., language or coding workshops), and involvement in educational trips or cultural visits organized by the school. Finally, the *Environment* dimension evaluates the availability of educational resources within the living environment, using indicators to examine access to public green spaces, parks, libraries, theatres, cinemas, museums, and sports facilities in the community. Specific details on these indicators are provided in Appendix S2 Appendix.

It is worth noting that our conceptual framework aligns with the new definition of EP proposed by ISTAT in the context of official statistics, which was presented at the “Fifteenth National Conference of Statistics” (Rome, 3rd-4th July 2024) and is currently being refined by a scientific commission. ISTAT adopts a multidimensional definition of EP structured in two main domains, which are educational resources and outcomes, where the former is made up of three subdomains referring to the family, school, and territorial context. In this regard, our proposal follows the three-dimensional resource structure in the definition of EO. However, two crucial differences should be highlighted: 1) ISTAT’s reference population includes children and young people aged 0–19, while our study focuses specifically on students aged 15–19 (which is considered a life period crucial for individual development); 2) The indicators proposed by ISTAT are derived from official statistics and thus have a territorial reference; instead, our indicators are designed to assess EP at an individual level, providing a more granular understanding of the phenomenon.

## 4 Data collection

The previously described conceptual model was implemented in a questionnaire that included information on family background and data on possible EP outcomes such as cognitive and non-cognitive abilities, aspirational ability, and lifestyles. A pilot survey of a convenience sample of students aged 15–19 residing in Naples and the province was then conducted to test the questionnaire and the conceptual model. From January to March 2024, a sample of high school students in their third to fifth years were surveyed. We undertook a rigorous selection process from the initial pool of 264 students surveyed: 73 students who did not pass an attention check were omitted from further analysis. This meticulous approach resulted in a final sample size of 191 students: 111 identified as female, 77 as male, and three as non-binary. This careful selection process helps maintain the accuracy of our analysis, supporting our findings. A descriptive comparison between included (N = 191) and excluded respondents (N = 73) was conducted using key socio-demographic, educational, and family background variables. Overall, the two groups display broadly similar distributions in terms of school performance. Some differences emerge in gender composition and in selected family background characteristics. However, these variations do not suggest substantial structural differences between the groups. Detailed descriptive statistics for excluded respondents are reported in Appendix S1 Appendix. The study was approved by the Ethics Committee of the University of Naples Federico II. Written informed consent was obtained from all participants before data collection.

The different sections of the questionnaire are dedicated respectively to collecting information on the three dimensions of educational opportunities (family, school, environment) and the individual characteristics of the respondents, as reported in Appendix S2 Appendix. All variables associated with the three dimensions of educational opportunities are binary, as respondents answer ‘yes’ or ‘no’ to the questions. The supplementary variables about individual characteristics are categorical.

## 5 Statistical analyses

The employed analysis strategy unfolds in multiple phases. Initially, a univariate statistical examination of all binary indicators is conducted to identify those with adequate variability. Exploratory factor analysis (EFA) assesses if the tetrachoric correlation matrix supports selecting a particular group of indicators to compute the scores for the three EO dimensions. Subsequently, confirmatory factor analysis (CFA) verifies the significance of the dimensional structure pinpointed by EFA. After determining the scores for the three dimensions by summing the indicators that effectively represent each dimension, hierarchical cluster analysis (HCA) categorizes respondents into groups exhibiting comparable patterns across the three dimensions. Lastly, supplementary variables are analyzed to discern the distinct group behaviours, considering personal attributes (such as gender), educational background (school type), academic achievements (grades in Italian and math, debts, failures), and parents’ educational level. For this purpose, graphical representations and statistical measures (like chi-square and Cramer’s V) are applied to evaluate the relationship between categorical variables and the cluster-derived group variable.

### 5.1 Factor analysis

Let **Y** be the matrix including the values of *K* observable indicators collected from a sample of *N* individuals. The main aim of factor analysis, or common factor model, is to achieve a more parsimonious and interpretable representation of **Y** by identifying a small number of underlying latent factors ξ that capture the joint meaning of subsets of indicators. More formally, the factor analysis model establishes a regression-type link function to connect the indicators with the latent variables, where the former represents the dependent variables and the latter the independents [45]. Thus, the model can be expressed as:


$$\begin{array}{c} \begin{array}{c} Y=\Lambda \xi +\varepsilon \end{array} \end{array}$$ (1)

where Λ is the matrix of factor loadings (or saturations) indicating the impact of ξ on the manifest indicators included in **Y**, and ε is the matrix of measurement errors, also called unique factors, reporting the proportion of variance in the indicators not explained by the common factors ξ. The model assumes that error components ε are random variables with zero means 𝐄(ε)=0 and diagonal covariance matrix. Additionally, they are assumed to be uncorrelated with the latent factors, hence Cov(Θ,ε)=0. Conversely, the sum of the squares of saturations that an observed variable presents on the ξ factors constitutes the proportion of its variance explained by the latent factors and is called communality. A high communality and a low unique factor are desirable for all manifest indicators, where their sum always equals 1.

Two main types of factor analysis have been proposed in the literature: exploratory factor analysis (EFA; [46,47]) and confirmatory factor analysis (CFA; [48]). The former is based on a data-driven procedure that extracts the number of common factors from the data without specifying the loading patterns between the observed and the latent variables. The latter is used in a confirmatory setting, where the number of latent factors and their associations with the manifest indicators are defined according to a substantive theory before analyzing the data. For more details about the differences between these two approaches, see [49–51], among others.

For both approaches, the starting point is the correlation matrix **S** between the observed variables, which is assumed to originate from their dependence on the latent variables. Note that different types of correlation matrices can be adopted at this stage according to the nature of the observed variables. Specifically, the Pearson correlation matrix is typically used with normally distributed indicators, whereas polychoric and tetrachoric correlations are preferred when dealing with ordinal and binary indicators, respectively.

In the EFA, manifest indicators can have non-zero correlations with all latent factors, so the first step involves estimating an initial factor structure. Model parameter estimation is based on the difference between the observed and predicted correlation matrices, where the latter can be expressed in terms of factor loadings, variability of the latent variables, and error terms:


$$\begin{array}{c} \begin{array}{c} \Sigma =\Lambda \Psi \Lambda^{\prime }+\Theta \end{array} \end{array}$$ (2)

with Λ indicating the matrix of factor loadings, Ψ the correlation matrix for the latent factors, and Θ the diagonal matrix of unique error variances. Several methods of factor extraction working on the proximity of the predicted and observed correlation matrix have been introduced. Herein, we rely on the *principal factor* method that exploits an ordinary least squares estimation procedure to minimize the following function:


$$\begin{array}{c} \begin{array}{c} F=tr[(S-\Sigma {)}^{2}] \end{array} \end{array}$$ (3)

where **S** and Σ are the observed and the predicted correlation matrix, respectively, and *tr* indicates the trace of the difference matrix. Note that a peculiarity of this method is that it operates on correlation matrices adjusted for the uniqueness factor. Accordingly, **S** and Σ present communality estimates, instead of 1s, on the main diagonal. The initial estimate of communalities is generally performed by calculating the squared multiple correlation *R*^2^ values for each observed variable, treating the others as independent variables in a regression model. Factor loading estimates are then obtained by minimizing the criterion in Eq (3) and the communalities in **S** are consequently updated. This iterative process is repeated until the change in the objective function value is below a certain threshold. For more details about this procedure, see [52].

Regarding the selection of the number of latent factors to retain, several statistical tools can be considered, which generally focus on the eigenvalues, namely the proportion of variance of the observed indicators explained by the latent factors. In particular, we consider two different criteria that are *parallel analysis* [53] and *very simple structure* [54]. The first approach involves generating random datasets with the same marginal properties as the observed data but without any underlying latent structure. The correlation matrix for each random dataset is computed, and the distributions of the corresponding eigenvalues are obtained. Factors in the actual data presenting an eigenvalue larger than the 95th percentile of the eigenvalue distribution from the random datasets are retained. The second approach, instead, selects the factor solution that best reproduces the observed correlation matrix assuming a very simple structure, that is each indicator loads on only one factor.

Once the factor structure is obtained, the interpretation of the latent factors becomes of core interest and usually requires a factor rotation to improve clarity. Indeed, in the initially extracted solution, indicators may load on multiple factors, yielding a factorial structure that is not clearly defined. In our study, we consider the *varimax* rotation method, among others, to obtain a more interpretable factor loading matrix. As an orthogonal method, the varimax rotation transforms the original factor loadings matrix, keeping the factors uncorrelated. Specifically, it maximizes the variance in the loadings for each factor across the indicators by increasing large loadings and decreasing the small ones so that few variables explain each factor.

The factorial structure that emerges in the EFA can be further tested by exploiting the CFA. Indeed, the empirical evidence from EFA guides the researcher in defining the constraints to place upon the factor structure before model estimation, which regard both the number of latent factors and the factor loadings (typically, each indicator is associated with only one latent factor). The CFA provides a series of useful model fit indices, including the Comparative Fit Index (CFI; [55]), the Tucker-Lewis Index (TLI; [56]), and the Root Mean Square Error of Approximation (RMSEA; [57]). Good model fit is usually defined by CFI and TLI values of approximately 0.90 or above and RMSEA values of approximately 0.05 or below. Moreover, the significance of factor loadings can also be verified.

### 5.2 Hierarchical cluster analysis

Let **X** be the data matrix containing the values of *P* variables for *N* individuals, represented herein by the individual factor scores obtained from EFA. Let **x**_*i*_ be the generic vector, including the values of the *P* variables for the *i*-th individual. Hierarchical cluster analysis allows for obtaining a sequence of nested partitions of the *N* observations by maximizing the within-cluster homogeneity and the between-cluster heterogeneity. Specifically, we exploit an agglomerative algorithm that begins with a partition of *N* groups, where each individual is a cluster, and progressively merges clusters until all observations are in a single group. At each step, the proximity between clusters is calculated using a linkage method, and the two closest clusters are merged. In our study, we use Ward’s linkage approach [58] that considers the cluster means and aims to minimize the sum of the squared distances of units from their cluster centroids. According to Ward’s method, the distance between two clusters $C_{1}=\{{𝐱}_{i}{\}}_{i\in C_{1}}$ and $C_{2}=\{{𝐱}_{i}{\}}_{i\in C_{2}}$, with sample size *n*_1_ and *n*_2_ respectively, can be expressed as:


$$\begin{array}{c} \begin{array}{c} d_{W}(C_{1},C_{2})=\frac{n_{1}n_{2}}{n_{1}+n_{2}}𝐝({\bar{𝐱}}_{C_{1}},{\bar{𝐱}}_{C_{2}}) \end{array} \end{array}$$ (4)

where ${\bar{𝐱}}_{C_{1}}$ and ${\bar{𝐱}}_{C_{2}}$ are the cluster centroids and $𝐝({\bar{𝐱}}_{C_{1}},{\bar{𝐱}}_{C_{2}})$ is their distance based on the Euclidean metric.

The output of a hierarchical clustering is usually displayed through a tree-like diagram, called *dendrogram*, which depicts the sequence of the nested partitions. The dendrogram branches show the hierarchical relationships among clusters, with units placed on one axis and the merging distance on the other. The dendrogram is particularly useful for selecting the number of clusters to retain, which involves choosing where to cut it. A widely used criterion is to cut the dendrogram at the level with the largest difference between cluster merges, indicating a significant change in within-cluster variance. For more details, interested readers can refer to [59,60], among others.

## 6 Results

### 6.1 Descriptive statistics

The indicators related to the three dimensions proposed to operationalize EO (family, school, and environment) are 30 and are all dichotomous. They represent the set of variables proposed for measuring EO and its components. They will be referred to below as *active variables*. The variables on personal characteristics are defined as *supplementary variables*, and Table 1 shows their distribution in the sample. Most individuals have no educational debt (85%), and a minority had to repeat the school year (14%). A large portion of the individuals (83%) have an adequate basic knowledge (>6) of Italian, while a smaller percentage reaches this level in mathematics (62%). About half of the individuals come from technical or professional institutes, and only a small group have parents with a high level of education (16% for fathers, 21% for mothers). Regarding parents’ occupational status, different patterns emerged for fathers and mothers, with the former mainly working as self-employed (30.4%) or teacher/employed professional (27.2%), while the latter mainly not working, retired, or their occupation was unknown (52.9%).

**Table 1:** Supplementary characteristics of survey respondents (15-19 years).

Variable	n	Percentage
SCHOOL DEBTS		
None	162	(84.8%)
One or more	29	(15.2%)
ITALIAN GRADE		
<= 6	32	(16.8%)
> 6	159	(83.2%)
MATHEMATICS GRADE		
<= 6	73	(38.2%)
> 6	118	(61.8%)
HELD BACK		
No	164	(85.9%)
Yes	27	(14.1%)
GENDER		
Other	3	(1.6%)
Female	111	(58.1%)
Male	77	(40.3%)
SCHOOL		
Other	28	(14.7%)
Technical-professional	94	(49.2%)
High school	69	(36.1%)
EDUCATION FATHER		
Low (ISCED 0–2)	79	(41.4%)
Medium (ISCED 3)	70	(36.6%)
High (ISCED 6–7)	31	(16.2%)
Do not know	11	(5.8%)
EDUCATION MOTHER		
Low (ISCED 0–2)	68	(35.6%)
Medium (ISCED 3)	69	(36.1%)
High (ISCED 6–7)	40	(20.9%)
Do not know	14	(7.3%)
OCCUPATION FATHER		
Executive, entrepreneur, etc.	12	(6.3%)
Teacher, employed professional, etc.	52	(27.2%)
Worker	36	(18.8%)
Not working, retired, unknown	33	(17.3%)
Self-employed	58	(30.4%)
OCCUPATION MOTHER		
Executive, entrepreneur, etc.	7	(3.7%)
Teacher, employed professional, etc.	41	(21.5%)
Worker	18	(9.4%)
Not working, retired, unknown	101	(52.9%)
Self-employed	24	(12.6%)

**Fig 1:**
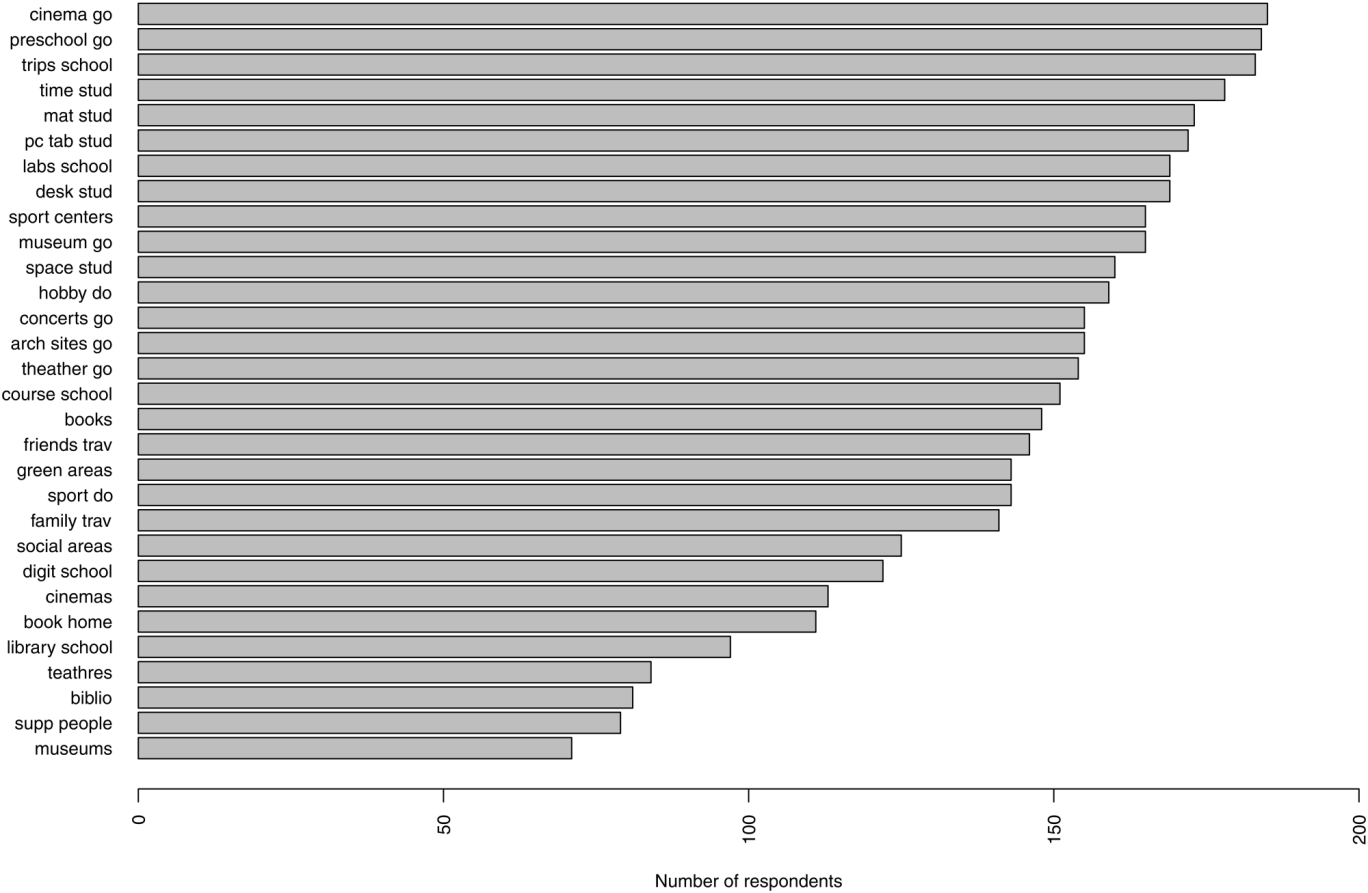
Endorsement frequencies for EO items from least to the most endorsed.

Fig 1 displays the response frequencies for all EO binary items, ranging from the least to the most endorsed. Among these items, *cinema go*, *preschool go*, and *trips school* exhibit minimal variation, with fewer than five responses in a category. Consequently, these three items have been excluded from subsequent analysis.

**Fig 2:**
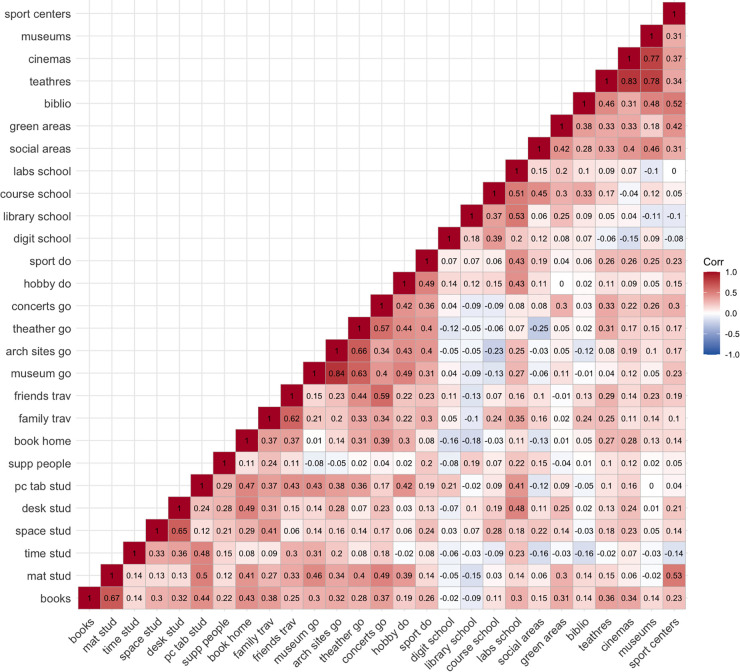
Tetrachoric correlations among the EO items.

Fig 2 presents the tetrachoric correlation structure among the 27 remaining indicators, enabling the examination of inter-item connections within each EO dimension (family, school, environment) and intra-item links across indicators from different dimensions. Within each dimension, correlations are generally positive, except for the *Family* dimension, where *supp people* has a negligible and negative correlation with *museum go* and *arch sites go*. To maintain homogeneity within the *Family* dimension, the *supp people* indicator is removed from further analysis. Negative correlations are observed between different dimensions, notably between *arch sites go* and *course school* (−0.24), and *social areas* and *theatres go* (−0.25).

### 6.2 Factor analysis

**Fig 3:**
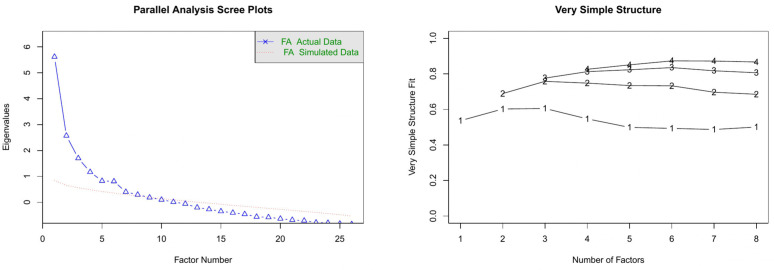
Optimal number of factors by parallel analysis (left side), and very simple structure (right side).

The EFA with *principal factor* method and *varimax* orthogonal rotation enables us to uncover the underlying dimensions within the 26 items chosen based on descriptive analyses. However, different criteria for selecting the optimal number of factors such as *parallel analysis* and *very simple structure* yield distinct solutions. While *parallel analysis* suggests 8 dimensions (an over-extraction), the *very simple structure* approach recommends 3 dimensions (see Fig 3). Consequently, we opt for a 3-dimensional solution in alignment with the questionnaire structure and underlying theory.

Table 2 outlines the factor loadings for each variable across the factors, their communalities, the proportion of variance each factor accounts for, and the total variance collectively explained by the factors. A three-factor model accounts for 41% of the total variance. The items *space stud* and *desk stud* have identical loadings on two factors and will be removed from future analyses. The items *time stud* and *digit school* exhibit low communalities and will also be excluded. The items *labs school* and *social areas* demonstrate relevant cross-loadings.

**Table 2:** Exploratory factor analysis results.

Item	Factor 1	Factor 2	Factor 3	Communalities
books	0.55	0.28	0.13	0.40
mat stud	0.63	0.14	−0.02	0.42
time stud	0.36	−0.15	0.08	0.16
space stud	0.30	0.15	0.30	0.21
desk stud	0.38	0.11	0.35	0.28
pc tab stud	0.66	−0.07	0.18	0.48
book home	0.49	0.16	−0.02	0.26
family trav	0.49	0.18	0.23	0.32
friends trav	0.52	0.20	0.00	0.31
museum go	0.71	−0.06	−0.08	0.51
arch sites go	0.71	−0.04	−0.12	0.52
theather go	0.72	0.08	−0.22	0.58
concerts go	0.63	0.27	−0.12	0.48
hobby do	0.55	0.02	0.16	0.33
sport do	0.45	0.19	0.15	0.26
digit school	−0.01	−0.02	0.33	0.11
library school	−0.10	−0.01	0.51	0.27
course school	−0.07	0.22	0.77	0.64
labs school	0.40	−0.05	0.78	0.77
social areas	−0.09	0.56	0.33	0.43
green areas	0.10	0.44	0.28	0.28
biblio	−0.04	0.59	0.16	0.37
theatres	0.20	0.84	−0.01	0.74
cinemas	0.20	0.76	−0.06	0.62
museums	0.05	0.82	−0.12	0.69
sport centers	0.24	0.51	−0.04	0.32
*% of Variance*	*0.19*	*0.13*	*0.09*	
*Cumulative %*	*0.19*	*0.33*	*0.41*	

Extraction method: Principal Axis Factoring. Rotation method: Varimax with Kaiser Normalization.

The arguments above suggest that the variables *space stud*, *time stud*, *desk stud*, and *digit school* should be excluded from subsequent analyses. The final results are presented in Table 3.

**Table 3:** Exploratory factor analysis results following item selection.

Item	Factor 1	Factor 2	Factor 3	Communalities
books	**0.54**	0.27	0.10	0.38
mat stud	**0.65**	0.13	−0.01	0.44
pc tab stud	**0.65**	−0.05	0.16	0.45
book home	**0.46**	0.15	−0.09	0.25
family trav	**0.48**	0.18	0.17	0.30
friends trav	**0.52**	0.21	−0.03	0.32
museum go	**0.72**	−0.08	−0.04	0.52
arch sites go	**0.72**	−0.06	−0.10	0.53
theather go	**0.76**	0.04	−0.18	0.61
concerts go	**0.64**	0.26	−0.14	0.49
hobby do	**0.61**	−0.01	0.23	0.42
sport do	**0.47**	0.18	0.18	0.28
library school	−0.10	0.00	**0.56**	0.32
course school	−0.06	0.24	**0.69**	0.54
labs school	0.40	−0.04	**0.90**	0.96
social areas	−0.08	**0.56**	0.31	0.42
green areas	0.10	**0.44**	0.27	0.28
biblio	−0.01	**0.59**	0.18	0.38
theatres	0.21	**0.84**	−0.01	0.75
cinemas	0.19	**0.77**	−0.07	0.62
museums	0.07	**0.84**	−0.14	0.72
sport centers	0.26	**0.49**	−0.03	0.31
*% of Variance*	*0.22*	*0.16*	*0.09*	
*Cumulative %*	*0.22*	*0.37*	*0.47*	

Extraction method: Principal Axis Factoring. Rotation method: Varimax with Kaiser Normalization.

**Fig 4:**
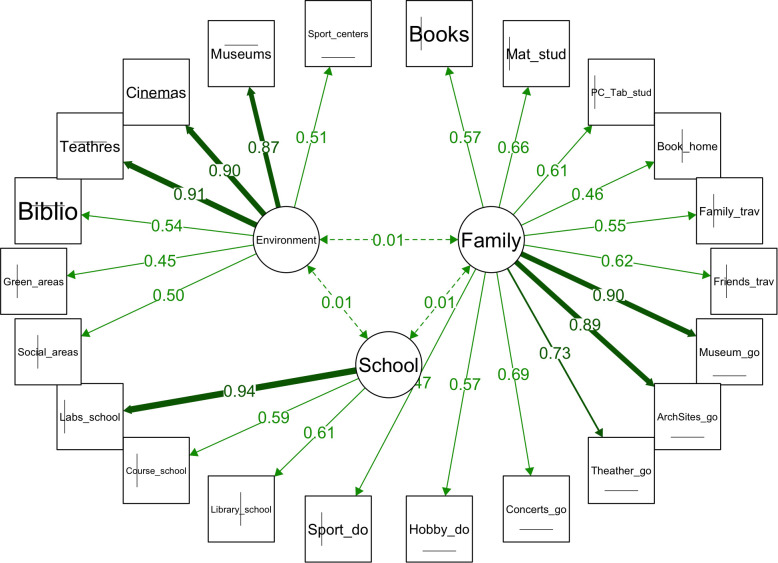
A conceptual framework of the EO dimensions (Family = Fml, School = Sch, Environment = Env).

The CFA uses model fit statistics and parameter estimates to evaluate the three-dimensional structure. Fig 4 illustrates a model demonstrating a satisfactory fit, enhanced by some extra connections among residuals suggested by the modification index. This is evidenced by the following metrics: the Comparative Fit Index is 0.95; the Tucker-Lewis Index is 0.95; and the Root Mean Square Error of Approximation is 0.04, with a confidence interval of 0.03 to 0.05. The significance of all factor loadings is verified in the Appendix S3 Appendix.

**Fig 5:**
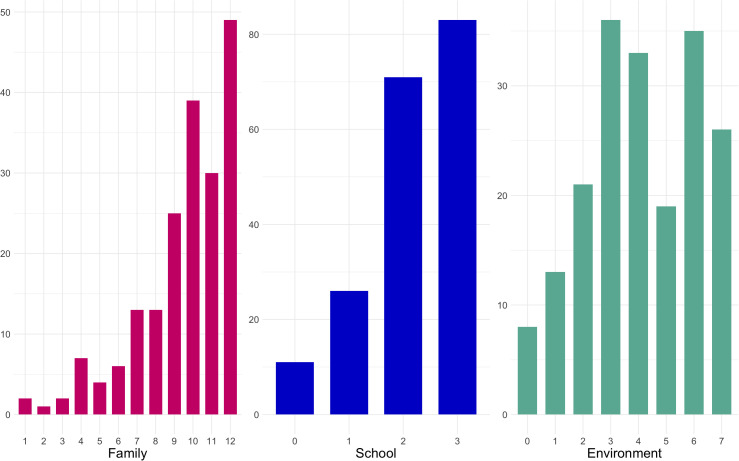
Sum scores of the EO dimensions.

After pinpointing the best markers for each EO dimension, we tally their scores by summing the elements’ values, considering their binary character. To assess the substantive equivalence between summed scores and factor scores, we computed factor scores for the three dimensions and correlated them with the corresponding raw summed scores; the correlations were high and near unity (Family = 0.97; School = 0.98; Environment = 0.96). This supports the use of summed item scores, a well-established psychometric practice given their stability, interpretability, and strong convergence with factor scores [61–63]. Fig 5 illustrates the spread of the three dimensions, with the variability distinctly tied to the varying count of markers composing them. Nonetheless, the three dimensions display a noticeable skewness, suggesting that many respondents exhibit elevated scores on all three EO dimensions.

### 6.3 Clustering

To explore EO further, we employ an HCA on the standardized scores of the EO dimensions plotted in Fig 5. This helps identify clusters of respondents who share similar profiles. We then characterize these profiles based on additional respondent characteristics outlined in Table 1 and defined *supplementary variables*. Given the exploratory nature of this approach, the resulting clusters are considered descriptive patterns emerging from the data, without implying fixed or definitive classifications.

**Fig 6:**
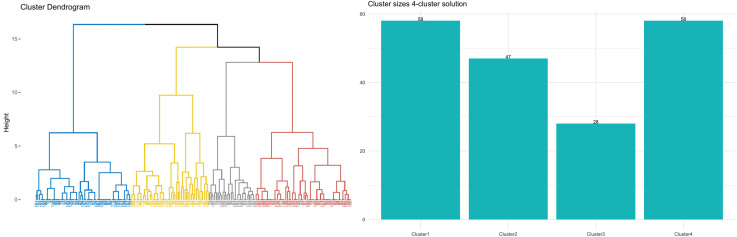
Dendrogram (left side) and clusters’ size (right side).

**Fig 7:**
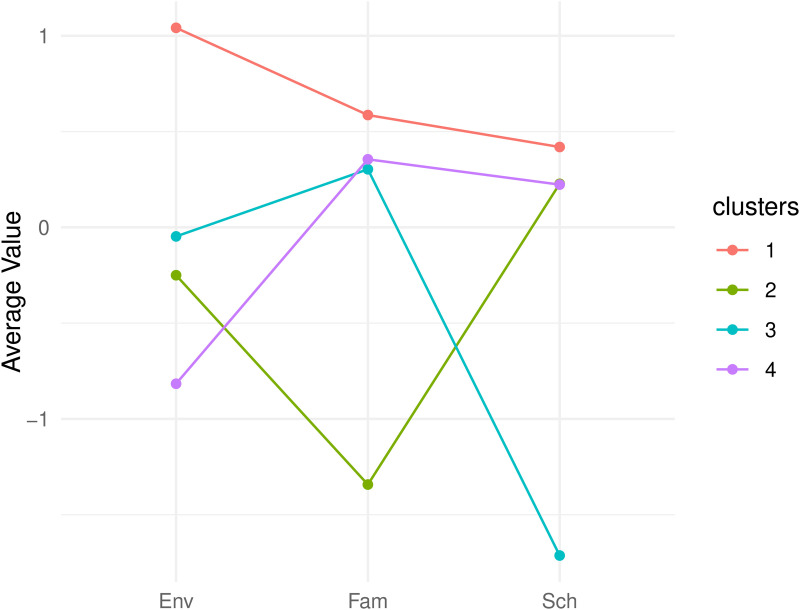
Clusters’ profile.

Results illustrated in Fig 6 indicate that the ideal cluster count is four, with each cluster nearly the same size. However, cluster 3 is an exception, containing fewer respondents. To acknowledge the exploratory nature of the clustering procedure and to address issues of stability and generalizability, we evaluated the robustness of the identified profiles using two widely recommended indices for determining the optimal number of clusters: the within-cluster sum of squares (WSS) and the silhouette coefficient (SC) [64,65]. Both indices supported a four-cluster solution as the most appropriate configuration. The results obtained from WSS and SC are presented in the Appendix S5 Appendix. Although a local peak was also observed for a five-cluster solution, the incremental improvement was minimal and would have resulted in the subdivision of an existing cluster. Given the relatively small sample size, such fragmentation would likely reduce cluster stability and hinder generalizability [66,67]. For these reasons, we retained the four-cluster solution, which represents the most stable, parsimonious, and interpretable structure for the present data and is consistent with methodological recommendations for exploratory clustering in small to moderate samples. Fig 7 provides a detailed profile of each cluster based on the average conditional values for the EO dimensions: cluster 1 is characterized by high opportunity levels across all three dimensions; cluster 2 is distinguished by limited opportunities in the *Family* dimension; cluster 3 is marked by reduced opportunities in the *School* dimension; cluster 4 is noted for its low opportunities in the *Environment* dimension.

To categorize respondents within each class based on supplementary variables, we examined the relationship between each supplementary categorical variable and the group categorical variable (cluster membership). The supplementary variables refer to several factors that the literature on the topic has highlighted as being related to EO. Investigating gender differences allows us to explore whether specific patterns of EO are more prevalent among males or females, given that inequalities in EO can contribute to gender disparities in academic outcomes. The type of school can also correlate with particular EO patterns. For example, EO within the family context can influence the choice of high school and subsequent career, and different school types reasonably offer various educational resources according to their curricula. Research has highlighted that EP, and thus a lack of EO, significantly affects students’ cognitive development. Hence, we expected that the differences in family, school, and environmental EO scores between the groups would also be reflected in academic performance, which we defined in terms of Italian and Mathematics grades, educational debits, and grade retention. On the one hand, academic achievement in core subjects like Italian and mathematics is a strong indicator of students’ overall academic capabilities. On the other hand, educational debits and experiences of being held back a grade reflect difficulties in the academic path. Finally, as thoroughly discussed in the theoretical section, disparities in EO are often related to socio-economic background, which includes parental education level and occupational status. Indeed, higher education levels are generally associated with better access to educational resources at home and participation in extracurricular activities. Similarly, parental occupation could influence EO not only through the availability of financial resources but also by providing access to high-status social networks and positive role models.

All the findings regarding the relationship between the supplementary variables and the cluster membership are presented in the figures included in Appendix S4 Appendix. The main results showed that parental education level, mother’s occupational status, Italian grades, gender, and type of school are significantly related to the identified groups. Conversely, educational debits, Mathematics grades, grade retention, and the father’s occupation do not significantly contribute to characterizing the cluster-derived groups. Specifically, cluster 1 consists of high school students whose parents have high educational qualifications, with a significant proportion of mothers employed in medium- to high-skilled executive or professional roles. Cluster 2 includes a larger share of students with low Italian scores, attending technical-professional institutes, and having parents with lower educational qualifications. In this group, most mothers are not employed. Cluster 3 primarily comprises female high school students whose parents have average educational qualifications, with a notable percentage of mothers working in medium-skilled professional positions. Lastly, cluster 4 is dominated by females with high Italian scores who attend technical or specialized schools, with parents whose education levels vary widely. This cluster has the highest proportion of self-employed mothers.

The interpretation of the clusters can be linked to the analysis of the literature on EP underlying the conceptual proposal presented in this paper. In particular, the characteristics of the first cluster show how family background, i.e., the presence of a family with a good standard of living (in terms of parents’ education and occupation) and a positive environment surrounding young people’s lives (school and local area), are strongly associated with high educational opportunities, which are reflected in good student performance. The crucial role of the family emerges even more clearly in the second cluster, which shows the presence of a vicious circle linking low levels of education and employment status of parents to poor educational opportunities for their children, which in turn affects their academic performance. Limited or scarce resources provided by educational institutions, including digital tools and extracurricular programs, affect student performance across all types of schools (high school or technical-professional), as shown in cluster 3. However, schools cannot bear sole responsibility for the shortcomings of the environment in which students live. The fourth cluster confirms that students with difficult family circumstances also find themselves living in an environment that does not contribute to healthy development and offers limited opportunities, such as public libraries, parks, and cultural institutions.

## 7 Discussion and conclusions

This study examines EP from both conceptual and measurement perspectives. We define EP in terms of family, school, and environmental EO and propose a set of indicators to measure each latent dimension in a sample of high school students. The results of the factor analysis confirm the three-dimensional structure of EO, consistent with the theoretical framework and research hypotheses. After selecting the most relevant indicators, we computed individual scores for each dimension and segmented the sample accordingly. The cluster analysis identifies distinct profiles of EO and their associations with contextual and individual characteristics, shedding light on factors that structure opportunities among students. Cluster 1 shows high EO across all domains and includes students whose parents have high educational attainment, providing access to cultural and social capital. In this group, highly educated parents—often with mothers employed in medium- to high-skilled occupations—appear to facilitate broader opportunities, supported by stronger economic resources derived from their labor-market positions. These students more frequently attend academically oriented schools and report stronger academic performance. Cluster 2, instead, is characterized by lower EO in the family domain and includes students from households where parents, particularly mothers, have low educational attainment or are unemployed. This pattern is consistent with the literature linking parental education and employment status to limited educational resources and lower academic expectations. These students predominantly attend technical schools and report lower academic performance, pointing to the cumulative impact of socioeconomic disadvantage. Cluster 3 comprises mainly female students who report reduced opportunities in the school domain. Despite this, they show good academic performance, and their parents are often employed in medium-skilled occupations, including a notable share of mothers. Finally, Cluster 4 consists primarily of female students with low EO in the environmental domain: despite relatively available family and school resources, the socio-territorial context offers limited opportunities outside school. Several considerations follow from these findings. First, parental socioeconomic status (SES) emerges as the most influential factor in structuring access to EO and, consequently, EP. This result aligns with established sociological research showing that parental SES affects the material and non-material resources available to children and adolescents. Higher SES is associated with greater access to opportunity-rich neighborhoods, better-resourced schools, and additional educational investments. Conversely, lower SES is linked to reduced opportunities, most visibly within the family domain, where constrained economic, cultural, and social resources may limit learning experiences, shape academic expectations, and restrict developmental opportunities beyond formal schooling [68,69]. With respect to our research design, the approach proposed in this study—using EO as a measure of EP—provides a valuable tool for understanding and assessing educational deprivation. While much previous research has focused primarily on direct manifestations of EP, such as test scores or educational attainment levels, our approach shifts attention toward the resources and opportunities that students can access as they grow. In doing so, it broadens the concept of education beyond cognitive outcomes to include social and emotional dimensions that are central to personal development. By explicitly considering the domains in which EO are produced and distributed, our framework emphasizes the multidimensional nature of EP and helps clarify how structural factors, such as family background, school quality, and environmental context, interact in shaping students’ opportunities and contributing to the production of EP. Moreover, this strategy supports a more nuanced assessment of EP, capturing not only whether students experience deprivation but also the extent and configuration of their disadvantages. The cluster results illustrate, for example, that even when EO are relatively strong in one or two domains, deficits in another can still be associated with EP. In this sense, structuring EO into three dimensions provides a comprehensive tool for identifying the presence or absence of opportunities and for pinpointing where interventions may be most needed. In addition, it offers policymakers a clearer view of the origins of educational inequality and of potential courses of action, particularly in the following areas: 1) Targeted interventions: by identifying domains where EO are lacking, policymakers can design focused measures, such as improving educational infrastructure in disadvantaged areas or strengthening school-based support systems; 2) Strengthening territorial resources: place-based policies can expand access to educational, cultural, and social services, especially where such resources are limited; 3) Structured interventions aimed at improving families’ socioeconomic conditions, including financial and employment support for low-income households, could help reduce inequality and limit the intergenerational transmission of disadvantage, including in the educational sphere. The study also has limitations that point to directions for future research. The sample is not representative, which limits the generalizability of the findings to the broader population. In addition, the analysis focuses on high school students in Naples and excludes both other geographic areas and adolescents of the same age who are not enrolled in school. Future work could therefore broaden the perspective by comparing students’ access to EO across different territorial contexts and by including out-of-school youth to provide further insight into the relationship between EP and the risk of school dropout. Finally, the analysis led us to discard eight of the thirty initial indicators because of limited discriminatory power and weaker measurement properties. This issue may be related to the relative homogeneity of the sample, suggesting that the proposed indicators and the resulting factor structure should be tested and refined using larger and more heterogeneous samples. Indeed, in line with the pilot nature of the study, the empirical analyses were primarily intended to explore the internal consistency and dimensional structure of the proposed framework rather than to provide a definitive validation of the measurement model. Moreover, the factor solution could be partially sensitive to analytical decisions, such as the criteria used to select the number of latent factors or the retained items. At the same time, the extraction and validation process followed established methodological guidelines, combining multiple retention criteria, rotation procedures, and confirmatory analyses to ensure internal coherence and interpretability. Therefore, the identified structure should be interpreted as a theoretically grounded and empirically supported configuration, obtained through rigorous methodological procedures, yet a sample-contingent approximation that warrants further validation.

Despite these limitations, this study provides an initial attempt to measure EP at the individual level through family, school, and environmental opportunities, with particular attention to adolescents. Future work could extend the full conceptual model proposed in Davino et al. [25,70] by further examining how socioeconomic background shapes EP and how restricted EO relates to developmental outcomes, including cognitive and non-cognitive dimensions such as socio-emotional skills, academic achievement, aspirations, and lifestyle. Overall, the practical contribution lies in offering stakeholders and policymakers a structured framework to identify where educational opportunities are lacking and to inform interventions aimed at reducing educational inequalities.

## Supporting information

S1 Appendix Characteristics of excluded respondents. (PDF)

S2 Appendix Questionnaire. (PDF)

S3 Appendix Confirmatory factor results. (PDF)

S4 Appendix Clusters’ profile according to the supplementary variables. (PDF)

S5 Appendix Determination of the optimal number of clusters. (PDF)
