# Supplementary material for: Assessing educational poverty: Insights into youth opportunities
Source: PLoS One. 2026 May 18;21(5):e0346156. doi: 10.1371/journal.pone.0346156 (PMC13183247; doi:10.1371/journal.pone.0346156)
Supplement: S1 Appendix — (PDF) [file pone.0346156.s001.pdf]

Appendix A. Characteristics of excluded respondents

Table 1. Supplementary characteristics of excluded respondents (failed attention check, 15-19 years)

Variable	n	Percentage
SCHOOL DEBTS		
None	60	(82.2%)
One or more	13	(17.8%)
ITALIAN GRADE		
<= 6	15	(20.5%)
> 6	58	(79.5%)
MATHEMATICS GRADE		
<= 6	25	(34.2%)
> 6	48	(65.8%)
HELD BACK		
No	63	(86.3%)
Yes	10	(13.7%)
GENDER		
Other	3	(4.1%)
Female	36	(49.3%)
Male	34	(46.6%)
SCHOOL		
Other	21	(28.8%)
Technical-professional	35	(47.9%)
High school	17	(23.3%)
EDUCATION FATHER		
Low (ISCED 0-2)	31	(42.5%)
Medium (ISCED 3)	20	(27.4%)
High (ISCED 6-7)	13	(17.8%)
Do not know	9	(12.3%)
EDUCATION MOTHER		
Low (ISCED 0-2)	22	(30.1%)
Medium (ISCED 3)	18	(24.7%)
High (ISCED 6-7)	16	(21.9%)
Do not know	17	(23.3%)
OCCUPATION FATHER		
Executive, entrepreneur, etc.	9	(12.3%)
Teacher, employed professional, etc.	21	(28.8%)
Worker	9	(12.3%)
Not working, retired, unknown	23	(31.5%)
Self-employed	11	(15.1%)
OCCUPATION MOTHER		
Executive, entrepreneur, etc.	5	(6.8%)
Teacher, employed professional, etc.	15	(20.5%)
Worker	5	(6.8%)
Not working, retired, unknown	43	(58.9%)
Self-employed	5	(6.8%)
