# Supplementary material for: Assessing educational poverty: Insights into youth opportunities
Source: PLoS One. 2026 May 18;21(5):e0346156. doi: 10.1371/journal.pone.0346156 (PMC13183247; doi:10.1371/journal.pone.0346156)
Supplement: S2 Appendix — (PDF) [file pone.0346156.s002.pdf]

Appendix B. Questionnaire

Table 1. Questionnaire

Label	Indicators
	**Family Dimension**
books	Do you have the necessary books for your school activities?
mat stud	Do you have all the school supplies (notebooks, drawing paper, calculators, dictionaries, etc.) you need to do homework and classroom activities?
time stud	Do you usually manage to dedicate the necessary time to study when you are at home?
	When you are at home:
space stud	Do you have a quiet space to study?
desk stud	Do you have your desk to study?
pc tab stud	Do you have access to a computer and/or tablet for studying?
supp people	Do you receive support and/or help with homework?
book home	Excluding textbooks, how many books are there in the house?
	During the year, do you have the opportunity to:
family trav	Travel with family
friends trav	Travel with friends
museum go	Visit museums, exhibitions
arch sites go	Visit archaeological sites, monuments
cinema go	Go to the cinema
theather go	Go to the theatre
concerts go	Attend music concerts
hobby do	Pursue hobbies in your free time (e.g., photography, etc.)
sport do	Engage in sports
	**School Dimension**
digit school	Do you use digital devices (tablet, PC, etc.) for educational activities at school?
library school	Are there libraries at school that you can use?
	Does your school offer you the opportunity to:
course school	Attend recovery or reinforcement courses?
labs school	Participate in extracurricular laboratory activities
trips school	Take part in cultural trips or organised outings?
preschool go	Did you attend kindergarten (preschool)?
	**Environment Dimension**
social areas	Are there enough places or spaces in your neighbourhood where kids your age can socialise?
green areas	In the area where you live, are there parks, gardens, or other public green spaces accessible within a 15-minute walk?
	Can you easily reach from the area where you live
biblio	Libraries
teathres	Theaters
cinemas	Cinemas
museums	Museums
sport centers	Sports centres (gyms, pools, etc.)
	**Supplementary variables**
Gender	Gender
School	Type of school attended
Italian grade	Grade in Italian
Mathematics grade	Grade in Mathematics
School debits	Having incurred school debts
Held back grade	Having been held back a grade
Occupation father	Father's occupation
Education father	Father's educational attainment
Occupation mother	Mother's occupation
Education mother	Mother's educational attainment
