# Supplementary material for: Assessing educational poverty: Insights into youth opportunities
Source: PLoS One. 2026 May 18;21(5):e0346156. doi: 10.1371/journal.pone.0346156 (PMC13183247; doi:10.1371/journal.pone.0346156)
Supplement: S3 Appendix — (PDF) [file pone.0346156.s003.pdf]

# Appendix C. Confirmatory Factor results

**Table 1. Factor loadings**

| LV | MV | Estimate | SE | t−values | p−values | ci.lower | ci.upper |
|---|---|---|---|---|---|---|---|
| Family | | | | | | | |
| | books | 0.530 | 0.104 | 5.106 | 0.000 | 0.327 | 0.733 |
| | mat stud | 0.622 | 0.126 | 4.936 | 0.000 | 0.375 | 0.870 |
| | pc tab stud | 0.658 | 0.125 | 5.251 | 0.000 | 0.413 | 0.904 |
| | book home | 0.507 | 0.093 | 5.475 | 0.000 | 0.326 | 0.689 |
| | family trav | 0.490 | 0.099 | 4.955 | 0.000 | 0.296 | 0.684 |
| | friends trav | 0.587 | 0.099 | 5.934 | 0.000 | 0.393 | 0.781 |
| | museum go | 0.625 | 0.103 | 6.097 | 0.000 | 0.424 | 0.826 |
| | arch sites go | 0.649 | 0.089 | 7.287 | 0.000 | 0.475 | 0.824 |
| | theather go | 0.811 | 0.077 | 10.591 | 0.000 | 0.661 | 0.961 |
| | concerts go | 0.748 | 0.083 | 9.030 | 0.000 | 0.585 | 0.910 |
| | hobby do | 0.617 | 0.101 | 6.123 | 0.000 | 0.419 | 0.814 |
| | sport do | 0.503 | 0.100 | 5.016 | 0.000 | 0.306 | 0.699 |
| School | | | | | | | |
| | library school | 0.610 | 0.127 | 4.819 | 0.000 | 0.362 | 0.858 |
| | course school | 0.591 | 0.131 | 4.496 | 0.000 | 0.333 | 0.848 |
| | labs school | 0.942 | 0.181 | 5.213 | 0.000 | 0.588 | 1.296 |
| Environment | | | | | | | |
| | social areas | 0.502 | 0.087 | 5.764 | 0.000 | 0.331 | 0.672 |
| | green areas | 0.450 | 0.105 | 4.283 | 0.000 | 0.244 | 0.656 |
| | biblio | 0.545 | 0.082 | 6.617 | 0.000 | 0.383 | 0.706 |
| | teathres | 0.914 | 0.039 | 23.426 | 0.000 | 0.837 | 0.990 |
| | cinemas | 0.905 | 0.045 | 20.195 | 0.000 | 0.817 | 0.992 |
| | museums | 0.872 | 0.046 | 18.851 | 0.000 | 0.781 | 0.962 |
| | sport centers | 0.508 | 0.117 | 4.345 | 0.000 | 0.279 | 0.738 |
