# Supplementary material for: Assessing educational poverty: Insights into youth opportunities
Source: PLoS One. 2026 May 18;21(5):e0346156. doi: 10.1371/journal.pone.0346156 (PMC13183247; doi:10.1371/journal.pone.0346156)
Supplement: S4 Appendix — (PDF) [file pone.0346156.s004.pdf]

Appendix D. Clusters' profile according to the supplementary variables

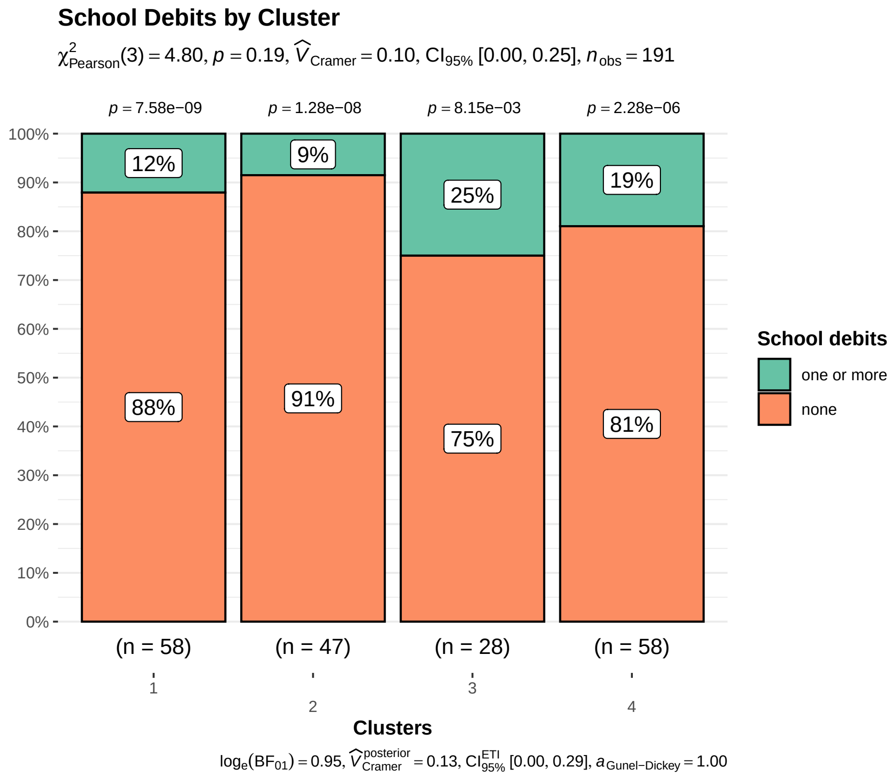

Fig 1. School debits

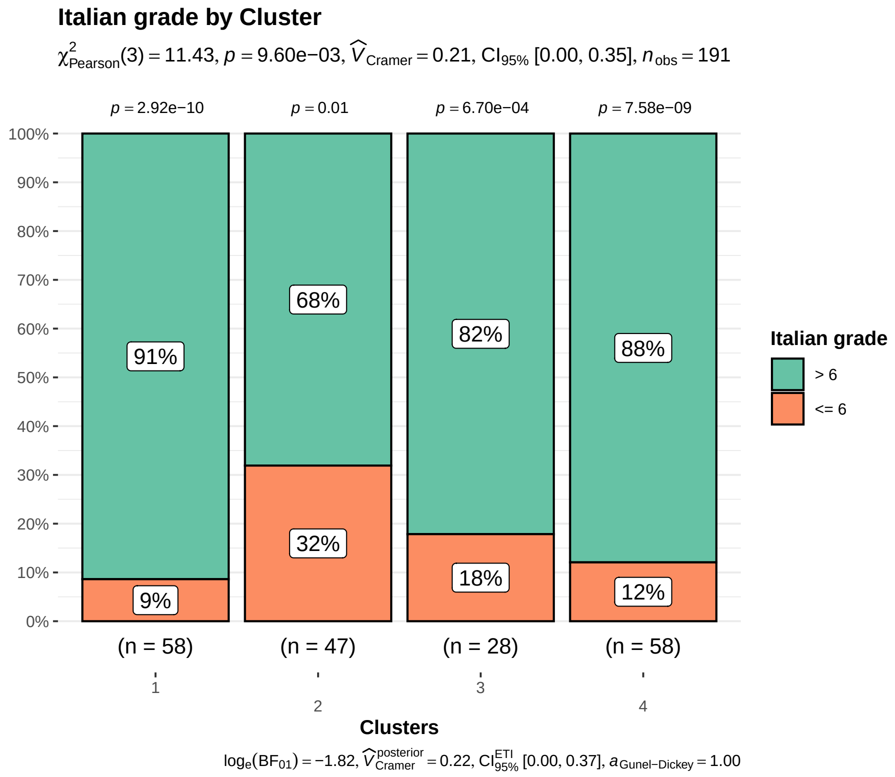

Fig 2. Grade in Italian

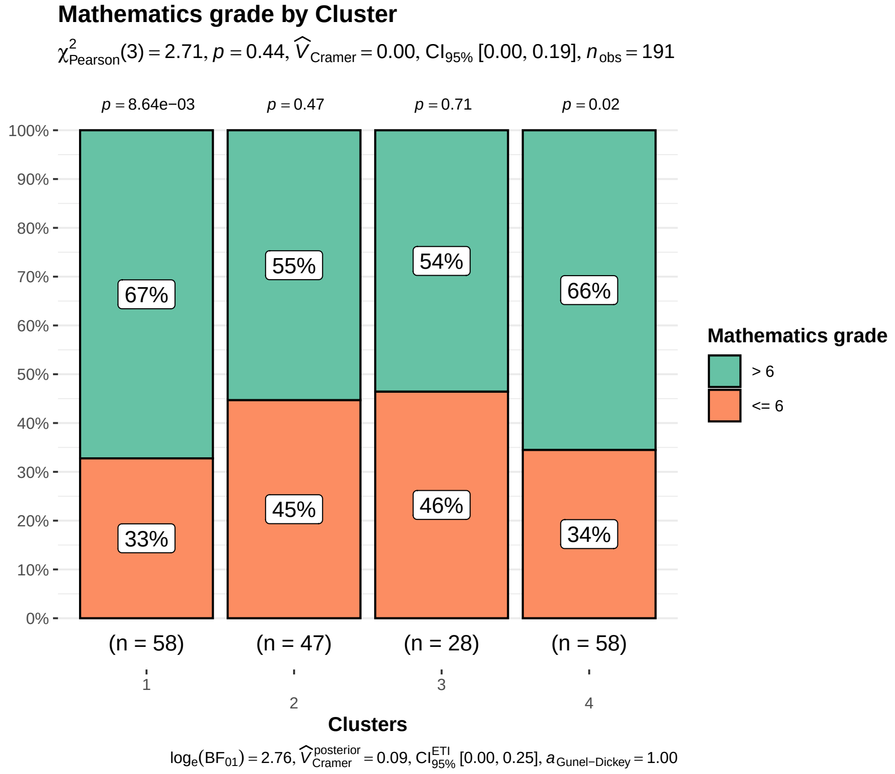

Fig 3. Grade in Mathematics

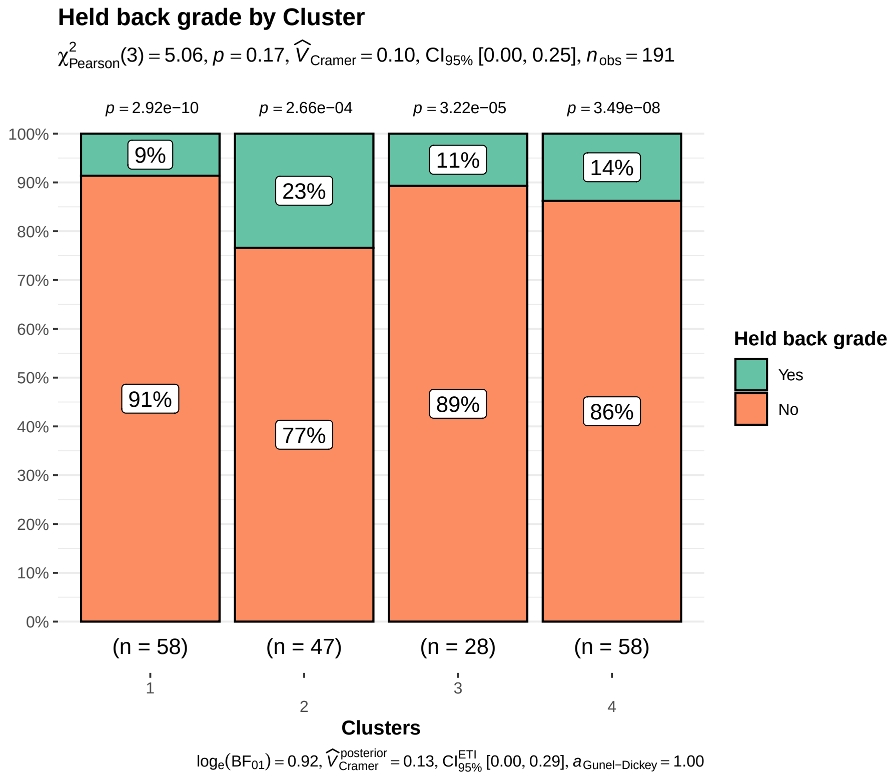

Fig 4. Held back a grade

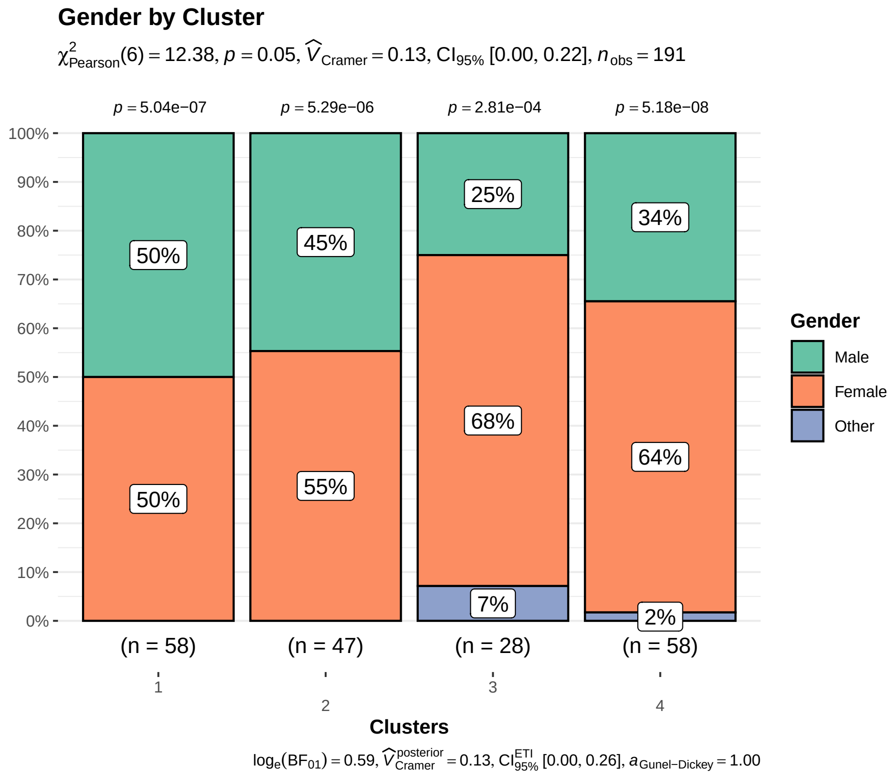

Fig 5. Gender

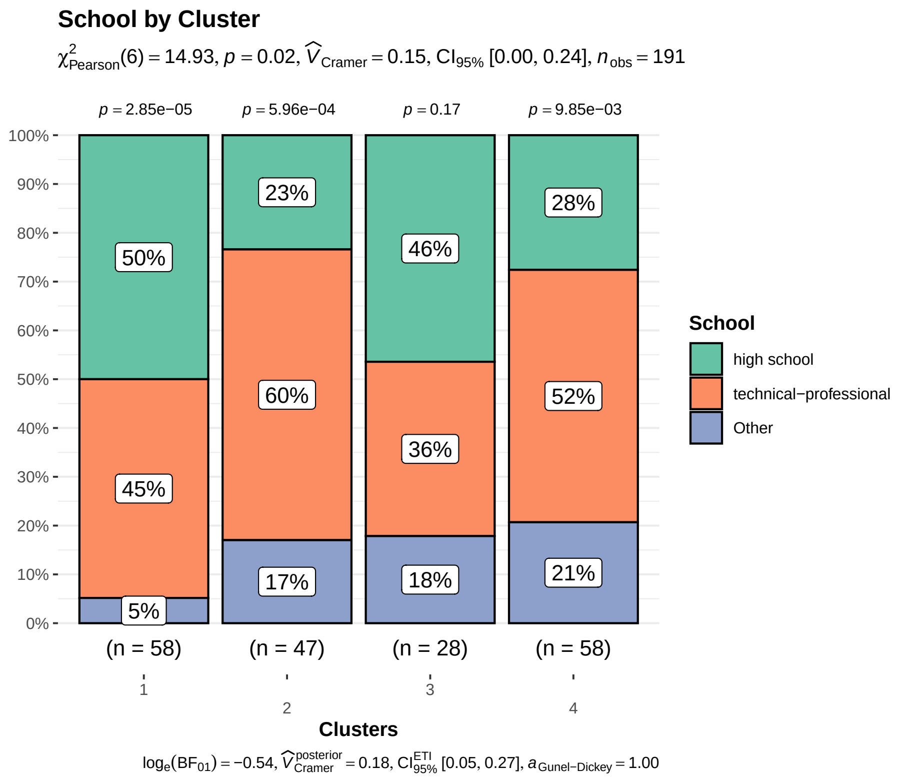

Fig 6. School

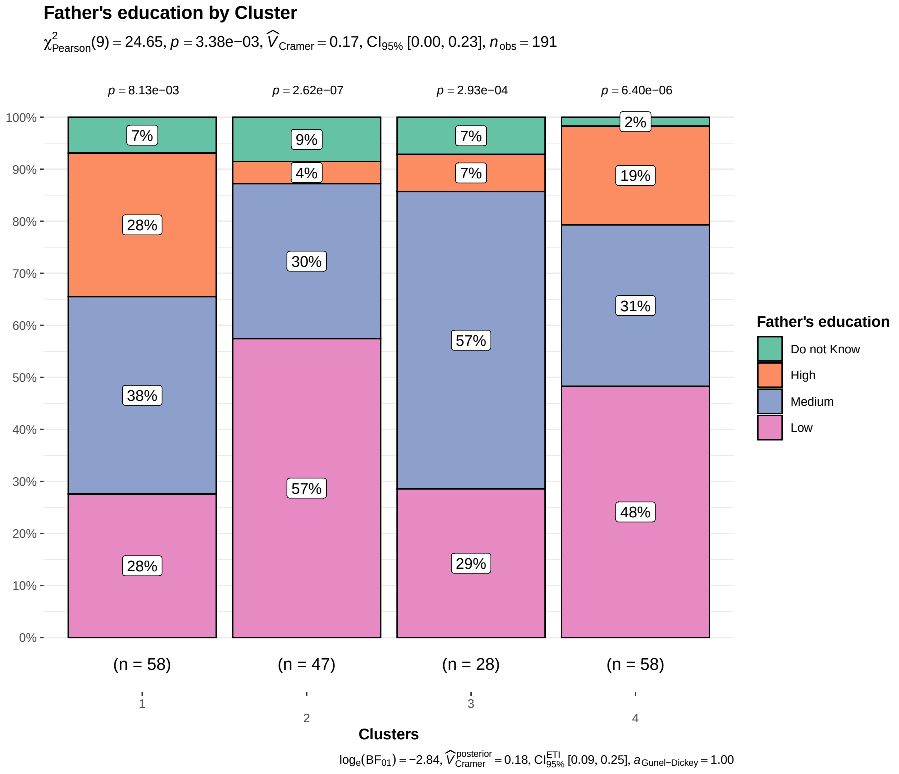

Fig 7. Father's education

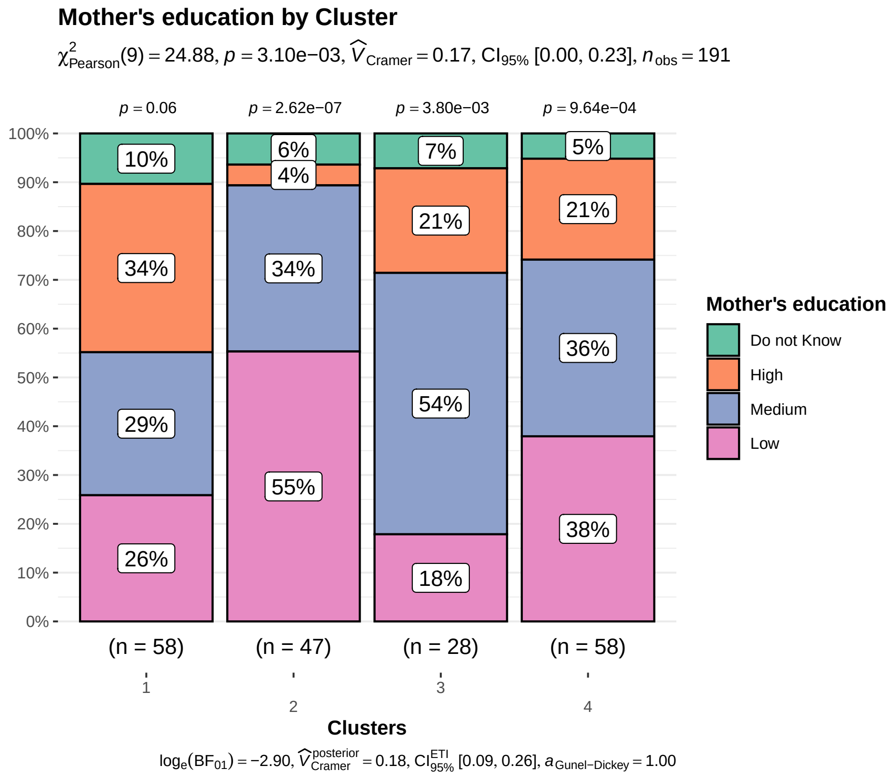

Fig 8. Mother's education

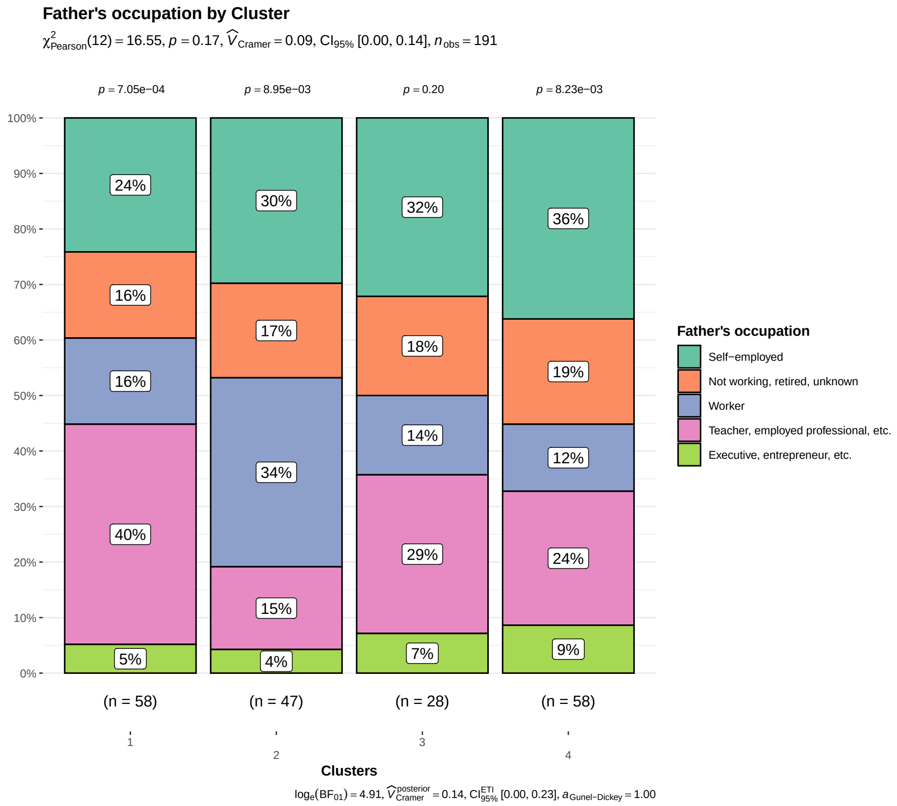

Fig 9. Father's occupation

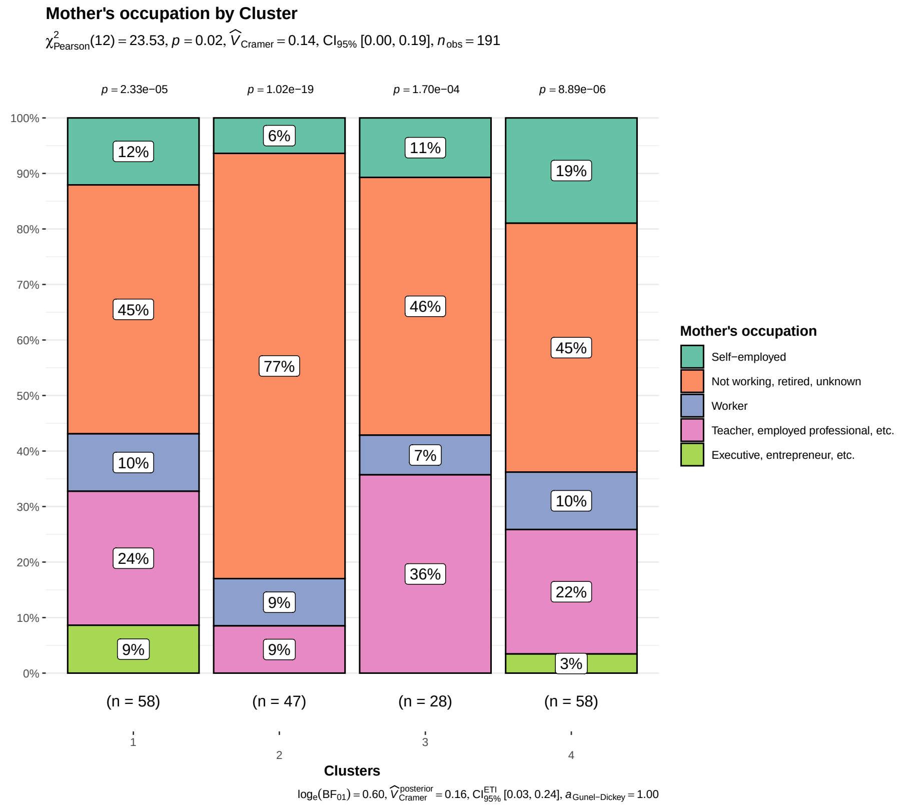

Fig 10. Mother's occupation
