# Supplementary figures and images for: Assessing educational poverty: Insights into youth opportunities
Source: PLoS One. 2026 May 18;21(5):e0346156. doi: 10.1371/journal.pone.0346156 (PMC13183247; doi:10.1371/journal.pone.0346156)

## Appendix E. Determination of the Optimal Number of Clusters

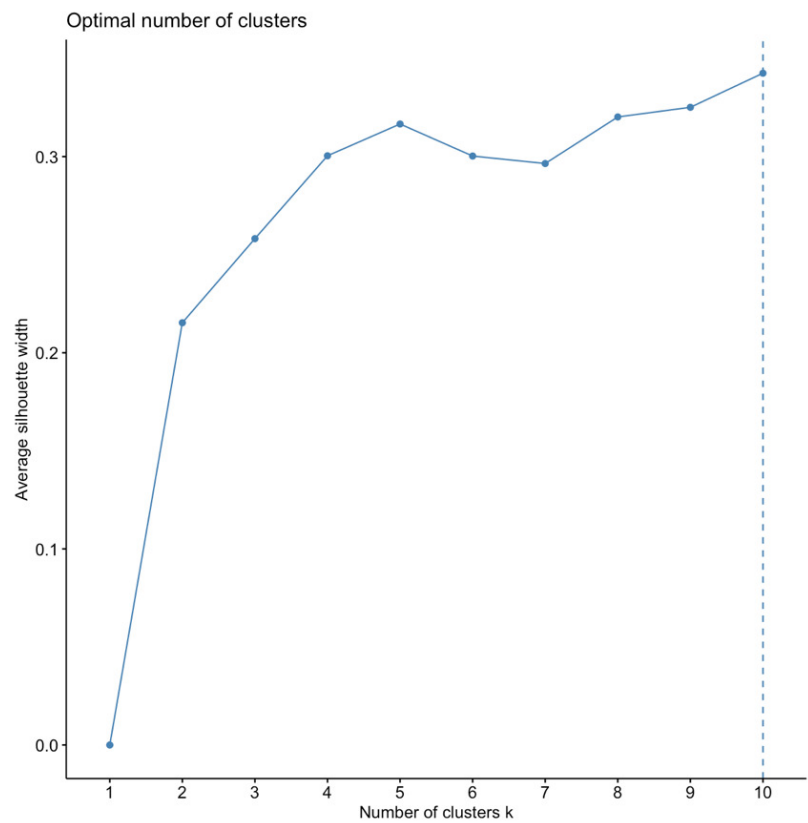

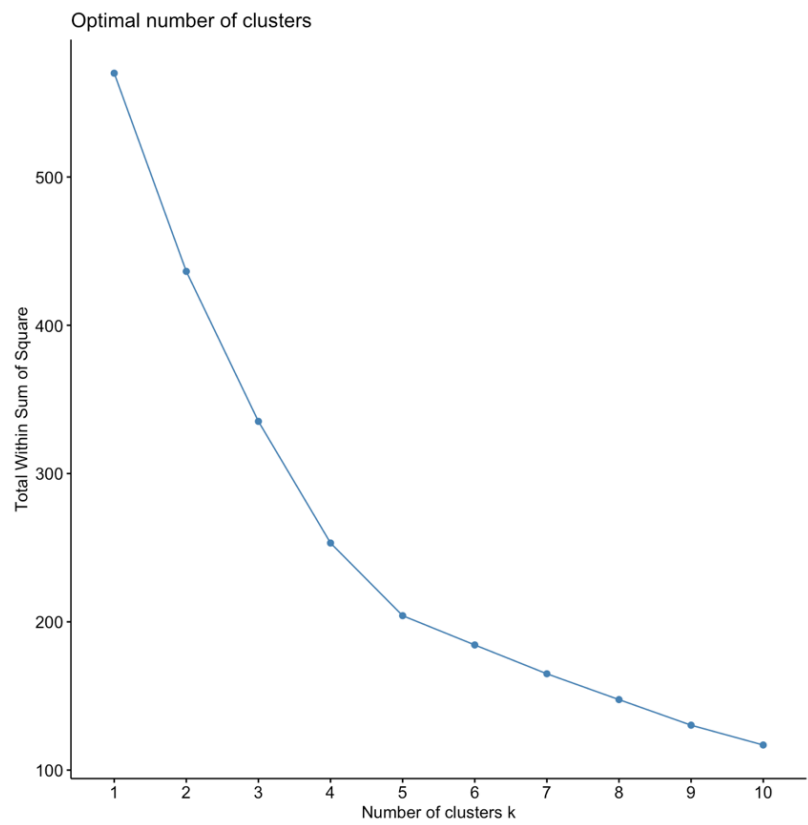

Supplement: S5 Appendix — (PDF) [file pone.0346156.s005.pdf]
